# A Courageous Report on the Management of Malnutrition

**DOI:** 10.3390/nu8100603

**Published:** 2016-09-27

**Authors:** Lucy B. Bollinger, Indi Trehan

**Affiliations:** 1 Department of Pediatrics, Washington University in St. Louis, One Children’s Place, Campus Box 8116, St. Louis, MO 63110, USA; lucy.bollinger@gmail.com; 2 Department of Paediatrics and Child Health, University of Malawi, Blantyre, Malawi

Globally, childhood undernutrition continues to be a major public health concern, with an estimated 165 million children classified as stunted and 51.5 million suffering from acute malnutrition. Childhood undernutrition increases the risk of childhood morbidity and mortality, impairs cognitive development and adult productivity, and may also increase the risk of certain diseases in adulthood. Approximately 875,000 deaths, or 12.6% of all deaths in children under the age of five, can be attributed to acute malnutrition [1]. Improving nutrition is a global priority, with the objective of eliminating world hunger by 2030 included in the 2015 United Nations Sustainable Development Goals [2].

Achieving this ambitious target will require improving and expanding treatment programs for moderate and severe acute malnutrition. Over the last 15 years, there has been a paradigm shift in the treatment of severe acute malnutrition, from inpatient management to home-based therapy with ready-to-use therapeutic foods (RUTF). Community-based management of acute malnutrition (CMAM) is now considered to be the standard of care for uncomplicated severe acute malnutrition (SAM) [3]. CMAM programs have been shown to be cost-effective and have been able to achieve recovery rates exceeding 90% with mortality in some settings of 5% or less. Much of the evidence for success in CMAM programs comes from programs conducted in the setting of controlled clinical trials or by large-scale implementation by nongovernmental organizations [4,5]. However, in order for CMAM protocols to have the broadest and most powerful impact, they must be scaled up and integrated into governmental health systems at national and local levels.

In their recent report in *Nutrients* entitled “Challenges in Implementing the Integrated Community-Based Outpatient Therapeutic Program for Severely Malnourished Children in Rural Southern Ethiopia”, Tadesse et al. critically examine the effectiveness of a community-based treatment program integrated into the local health care system in southern Ethiopia [6]. Study personnel unaffiliated with the treatment program visited health posts in the districts being studied to collect data on how closely the Community-based Outpatient Therapeutic Programs (C-OTPs) were adhering to national and World Health Organization (WHO) guidelines for the management of SAM. Their findings were sobering: only 39% of SAM enrollees recovered and were discharged from the OTP according to national guidelines. Of those children who did not recover within eight weeks, only 1.2% were appropriately transferred to inpatient care. Additionally, only one in three children had adequate and uninterrupted RUTF provision during the entire duration of treatment and one in five was given antibiotics on admission to the program. To report these markedly suboptimal results is an act of courage and the authors are to be commended for their work and effort in advocating—in a data-driven way—for optimal care to malnourished children in Ethiopia. These results highlight the challenges of scaling up and integrating CMAM into the lowest levels of the health system, as has been done in Ethiopia.

Studies of CMAM programs which specifically evaluate scaled-up, operational treatment programs not affiliated with a non-governmental organization (NGO) are limited. In Ethiopia, other groups evaluating the C-OTP program have reported recovery rates as high as 79% in a much smaller study in southern Ethiopia and 62% in northern Ethiopia [7,8]. Both of these studies were retrospective cohort studies using the patient cards kept by the local C-OTP. A review of government-run CMAM programs in Malawi, Zambia, and Ghana showed national recovery rates at outpatient therapeutic centers of 90%, 80%, and 73%, respectively [9]. In Burkina Faso, a pilot CMAM program run by the Red Cross was effective, with a recovery rate of 86.5% for SAM; however, efforts to transition and integrate the program into the existing national health care infrastructure were less successful: a lack of resources and advocacy were barriers to sustainability [10].

Among these studies, the prospective design and verification of anthropometric measurements, RUTF distribution, and other metrics by independent data collectors is unique to Tadesse’s important study in *Nutrients*. This rigorous design allowed for verification of the accuracy of recorded C-OTP data and did not limit the investigators to the information collected on patient cards. The authors are to be commended for critically examining the C-OTP program and for disseminating their results, for it is only through critical analysis that problems can be identified and solutions to these problems developed. For example, the authors identify a need for improved distribution of supplies such as RUTF and antibiotics, and for strategies to reduce sharing and selling of RUTF as specific areas to target for improvement. However, simply identifying this information is not enough: the next step must be using this knowledge to make substantive changes. Then, the process of evaluation can begin anew.

Today, the question facing home-based therapies for acute malnutrition is no longer whether they are effective; it is how to best expand and integrate CMAM into the primary health care system so that every child has access to potentially life-saving care. The evidence presented by Tadesse et al. [6] shows that doing this while maintaining the high recovery and low mortality rates which we know are possible will be a challenge. However, it is one that can be met in part by following the example of these authors and continuing to identify and share our failures along with our successes.
